# Destruction of the Trophic Function as an Element in Determining the Localization of Infection, Etc.

**Published:** 1888-09

**Authors:** Bayard Holmes

**Affiliations:** 125 State Street


### The Medical Journal and Examiner.

VOLUME LVII. SEPTEMBER, 1888. NUMBER 3.

### ORIGINAL COMMUNICATIONS.

### DESTRUCTION OF THE TROPHIC FUNCTION AS AN ELEMENT IN DETERMINING THE LOCALIZATION OF INFECTION, ESPECIALLY IN THE JOINT AND BONE DISEASES.

BY BAYARD HOLMES, M. D.

The inflammatory diseases of the joints, like most diseases elsewhere, depend upon the presence of a parasite. In a diseased individual not all the joints are affected. The susceptibility of a particular joint must depend on some anatomical, physiological, or pathological peculiarity. It is my purpose to call attention to one of the minor causes, which in a few cases seems to result in a point of least resistance, and thus determine infection.

In quite a large number of cases of tubercular joint-disease, where excision has, and where it has not, been performed, the patients have ultimately developed symptoms of posterior spinal sclerosis. This phenomenon is capable of various explanations. We might think that the tubercular infection was secondary to syphilis, which is often the primary cause of the sclerosis. Allowing this, there are cases of non-inflammatory destructive joint-disease occurring early and late in *locomotor ataxia* which are not so accounted for. They are thought to depend on the destruction of so-called trophic centres in the cord. It may not be amiss to review the proofs of the existence of the trophic function of the nervous system.

(1) The presence of an adequate supply of blood is recognized by all as necessary to the vegetation of a part. It seems, also, that a part which does not perform its inherited function soon falls into a condition of atrophy. Besides these common forms of altered growth, there occurs after the injury of a nerve trunk, or after its section, a peculiar form of perverted nutrition, which is observed in the skin as a glazing, in the muscles as a degenerative atrophy, and in the bones and joint-ligaments by corresponding involuntary changes. Such changes sometimes follow injury of the cord, and in these cases there is no interference with other nervous manifestations. From a consideration of these and similar cases, and experiments on animals, the idea of a trophic function has been deduced.

If the nervous system has a trophic function, it may be exercised through the sensory or motor nerves, or through a separate set of nerves. These nerves may derive their trophic impulse either from nerve centres which perform this office alone, or from centres having a mixed function, *i. e.*, from motor or sensory centres.

In order that the trophic function of a part may be abolished, and all other nervous phenomena remain undisturbed, it is necessary to pre-suppose one of the following conditions:

1. Unmixed trophic centres and mixed nerves.

2. Mixed centres and unmixed trophic nerves.

3. Separate trophic centres and separate trophic nerves.

Let us consider this subject from an embryological, anatomical, physiological, and a pathological standpoint.

*a.* That some influence is necessary to the division and growth of a cell, is seen in the ovum, which requires impregnation. Balfour has shown that the nerve-fibres are outshoots of the central nerve-cells. If this is always the case, our first and second suppositions are absurd.

*b.* Anatomy has little to offer. The best staining methods have so far failed to discover any difference in the appearance of functionally different nerve-fibres (Schwalbe). At most, the sum of all the nerve-fibres which enter or leave the cord is greater, by far, than the number which pass from the cord into the medulla.

*c.* Physiology only demonstrates that there is a separate trophic function in some animals, at least, and in some localities, which may be interrupted without disturbing sensation, motion, or special sense in the part. Thus the section of a certain portion of the fifth nerve determines a haziness and then destruction of the cornea on the same side without any interference with sensation. Section of the nerves surrounding the second cervical ganglion in cats results in the loss of all the hair over definite areas of the ears and head. This takes place uniformly in the course of a very few days. Microscopical examination demonstrates the degeneration of the nerves leading up to the hair bulbs, which are represented only by spots of pigment. These investigations have been carried out very completely and carefully by Joseph, and he is led to conclude that there are not only trophic centres but also trophic nerves.

Section of the cord through definite segments does not seem to interfere with the trophic function of the paralyzed or anæsthetic side. Destruction or removal of a portion of the motor area in the brain does not result in trophic disturbance in the paralyzed area. There are also other physiological demonstrations which lead to the conclusion that the trophic nerve-centres are not situated in the brain.

*d.* Pathology presents a great array of facts which it is very hard to explain without the existence of the trophic function. In *polyomyelitis anterior acuta* certain multipolar nerve-cells in the anterior gray horns are uniformly degenerated, as well as a portion of the anterior nerve-roots. The same pathological condition is present in progressive muscular atrophy. In injury or destruction of a large nerve-trunk it is a common thing to see glazing of the skin, loss of hair, defect in the nails, anchylosis of joints, atrophy of muscles, and disappearance of the *panniculus adiposus*, which conditions do not occur in loss of function through the destruction of the cerebral motor-area.

After compression of the cord by a displacement of the vertebræ, when death does not immediately occur, a number of very peculiar trophic disturbances are observed. Bed-sores occur in different places, depending upon the seat of the destruction. The muscles waste, and the resulting connective-tissue contracts, and greatly deforms the limbs. Repair goes on very slowly, or not at all, after the slightest injury. The circulation is modified by changes in the blood-vessels.

After puncture of the cord with a sharp instrument, in the region of the second lumbar vertebra, complete destruction of the ligaments of the knee-joint has been observed to follow in the remarkably short space of eight days (Sonnenberg).

In that peculiar and rare tropho-neurosis which sometimes affects one side of the face after the acute exanthemata and diphtheria, termed Progressive Facial Hemiatrophy, we have what seems to be a destruction of the trophic function of the fifth nerve, like that so frequently produced artificially in the laboratory.

These phenomena are easily reconciled if we admit the existence of trophic centres. The embryology of the lower animals indicates that the nerve is simply a continuation or an outgrowth of the nerve-cell, and so the conclusion is forced upon us that there are also trophic nerves. This is further demonstrated by the experiments of Joseph. Then I believe that the conclusion is inevitable, which was stated in the third proposition at the beginning of this section, *i. e.*, "*There exist separate trophic centres and separate trophic nerves.*"

In regard to the location of these trophic centres, it is safe to say that some of them are situated in the anterior gray horns; others are found in the posterior gray horns or in the ganglia which are located on the cranial and spinal nerves.

(2) In volume 8 of the *American Journal of Medical Sciences*, Dr. J. K. Mitchell called attention to some forms of "rheumatism" which he supposed to be due to a disease of the cord. Acting on this idea he recommended and practiced a very rational mode of treatment: In one case, cupping the spine where the nerve-trunk which supplied the affected limb entered the cord; in another, using immobilizing apparatus. At least one case was associated with locomotor ataxia. The whole matter was forgotten for many years until Charcot gave his classical lectures from which the disease has taken its name.

There has been a lively discussion as to the cause of this disease. Surgeons have paid very little attention to the evidence of the neurologists, and have always looked upon the existence of a neuropathic joint-disease with a condescending distrust. Therefore, all the cases have been described by the neurologists to whom the patients had previously come with evident nervous symptoms. This explains why so few cases have been observed as the initial symptom of disease of the cord.

Only a few post-mortem examinations have been made. A great majority of the cases have been reported many years, and by those who practiced very imperfect methods of examination. On account of these imperfections in the literature, many of the questions which might perhaps be answered by the recorded cases, if more accurately observed, must still be considered under judgment.

We have considered the evidence, and conclude that there are trophic nerve-centres and trophic nerves. It seems that those that control the nutrition of the skin are located in the posterior gray horns of the cord, while those that control the nutrition of the bones and joints are located in the anterior gray matter, or in the ganglia on the posterior nerve-roots. When these latter trophic cells are destroyed, either by mechanical violence or in the process of disease, the joint or bone or other tissue supplied by them fails in some way to vegetate. The cells of the joint structure undergo involution, degeneration, necrosis, and are more or less completely removed. If the destruction of these trophic cells is incomplete, the consequent changes in bone or joint fall short of complete destruction. There is no reason why such trophic changes in a joint may not originate either from a focal circumscribed disease in the gray matter, or from the involvement of the trophic centres in a system sclerosis.

It is well known that the initial symptoms of posterior spinal sclerosis are very different in different cases, which ultimately resemble each other closely. Thus, in one case — considering now the condition of the sensory nerves alone—there is no inco-ordination. The patient stands and walks well both with the eyes open and with them closed. He is able to recognize slight differences in weight, but the delicate or superficial sensibilities, as determined by the anæsthesiometer, are much reduced or entirely wanting. In another case where sensibility to light touch is unimpaired, co-ordination of motion is lost more or less completely, except as controlled by the eye, thus showing the destruction of the deep sensibilities—that is to say, in the first case there is loss of cutaneous sensibility, in the second, of muscular sensibility. Similar comparisons may be made in regard to the thermal sense, and the cutaneous and deep reflexes. When these cases have advanced far enough, the different forms of sensibility are completely or almost completely destroyed, and cases which were at first scarcely recognizable as the same disease become almost identical. In one case the deep muscular sense is first lost; in another, the sense of pain; in another, the sense to light touch; in another, the thermal sensibility; in another, deep reflexes; in another, the superficial reflexes; and in still another, the condition of perspiration. Then follow the remaining sensibilities one after another, as the centres in the cord which elaborate these impressions are destroyed.

In the same manner, we may suppose that the trophic cells are first destroyed, and that the resulting joint-disease is the first symptom of sclerosis. When the other trophic centres are unaffected, the other parts of the limb are unaffected.

We might expect that a joint on one side being affected, the centres of the trophic influence of the other, lying so near in the opposite gray horn, would be likely to suffer also. This appears to be the case. Out of one hundred and ten cases which form the basis of this study, symmetrical joints were attacked twenty-six times or in about one-fifth of the cases, while in only nine cases were asymmetrical joints affected (Rotter).

In support of this hypothesis there are many interesting clinical observations. Joint-diseases have already been recognized as the result of contusion, section, puncture, gunshot wound, and compression of the cord. In syringomyelitis, joint-diseases exactly resembling those of tabes have been observed. In the growth of a tumor of the cord, joint-disease has suddenly appeared.

Dissection of affected joints shows a remarkable disappearance of the nerves.

Experimental joint-disease has not been produced.

Adamkiewicz has demonstrated a very complex double circulation for the ganglion cell, which may have some influence in determining its destruction by thrombosis or embolism. It is, briefly, an enlargement of a little artery into a hollow sack, in which the cell is found. This sack is perforated in three or four places for the passage of the poles of the cell and for the vein. Thus the ganglion-cell is bathed on all sides by arterial blood. The venous blood passes to the interior of the cell and there surrounds the nucleus as a bladder-like cavity. It gives off one or two branches, which perforate the arterial sack and empty into the nearest vein. An idea of the arrangements of the blood-vessels may be obtained from Figure 155, Volume II of Quain's Anatomy, 1882. The drawing was made for a different purpose.

*The Neuropathic Joint-Disease* occurs in two forms, the early or mild form, and the later or destructive form. Only a few cases of the former have been observed.

Perhaps the most important symptom of the disease is the sudden, painless, swelling of the joint. In a few hours, or, rarely, in the course of a day or two, the joint fills with a serous exudate, which is in some cases mixed with a small quantity of blood. At the same time, or after a few hours or a day, the soft parts about the joint become swollen, but the skin retains its normal color. In the case of the knee, the swelling extends to the ankle below and to the middle of the thigh above. Over this whole area the engorged veins show through the skin. The swelling resembles a myxœdema, and it is probably also due to a central nervous lesion. The swelling of the joint is not always the first symptom recognized. There are a number of cases in which painless crepitus preceded swelling; and, in a few cases, spontaneous dislocation of the joint was the first recognizable symptom. The course of the disease is as painless as its beginning. When the knee is affected the patient goes about with the swollen and crepitating joint until the deformity and destruction have rendered the limb mechanically useless. The patient complains of the deformity rather than of the functional destruction. Exceptionally the disease begins with lightning pains in the limb, which are soon followed by the joint destruction.

Very often the knee-joint is unexpectedly dislocated without any assignable cause. Then examination shows the loss of the ends of the bones, or of the ligaments. Injury of the blood-vessels has caused alarming and almost fatal hæmorrhage into the connective-tissue space or into the joint.* These injuries are ordinarily painless. The first evidence the patient has of the mischief is the diminished mobility of the joint, or its swelling or discoloration. The resulting deformities are sometimes *genuvalgum* or *genuvarum*. When both knees have been affected, one has presented one deformity and one the other. In spite of such a condition the patient is able to go about on the waggling and creaking joint on account of the coincident anæsthesia. When the deformity has resulted in the total loss of the function of the joint, the application of splints puts the patient on his feet, and enables him to go about with stiff legs. Backward luxation of the tibia has been observed in fewer cases than would be expected from the frequent flail-like condition of the joint.

Very few cases have been observed in the mild or initiating stage. They begin as painless swellings of a joint without loss of function, and may continue in that condition for years. There is evident reason why they have been so rarely observed. They are painless, and occur in patients who are not under the care of physicians for other diseases. Hence they are considered trivial by the sufferer, and the advice of a physician is not sought. The profession, too, has paid little attention to neuropathic disease of the joints. There is every reason to think that they are more frequent than reports would indicate.

The number of neuropathic joints that become infected with the pus-microbes is not small. Out of Rotter's 112 cases seven suppurated, and in three cases no source of the infection could be assigned except auto-infection.

Blum (Charcot): Two years after the discovery of a serous fluid in the shoulder and knee joints by puncture, the case came to section, and they both contained pus.

Boucheret: Spontaneous dislocation of the hip-joint. Came to section on the thirty-first day of the disease. The joint was found infected.

Ponfick: Hip-joint. Unexplainable infection.

When we consider that the average duration of this disease is less than two years, the infection of seven or even of three cases by metastasis out of 112 cases is certainly a remarkably large percentage. While the anatomical changes in the rarer or milder form are less extensive, they are the same in kind, and the fact that the disease then extends through years, increases the chance of infection very materially. This is especially the case in tubercular infection, which is so slow in producing recognizable lesions. I am not able to find a case of tubercular joint-disease which occurred in a joint which was previously recognized to be the seat of neuropathic degeneration. In almost any table of a hundred cases of tubercular joint-disease one or two can be found that manifested signs of *posterior spinal sclerosis* within a few years after the

* The hæmorrhage which is sometimes found in the joint is due to mechanical destruction of the degenerated tissue containing a little blood-vessel.

The luxations are pure destruction luxations, and in no way due to the ordinary mechanical causes. This destruction may be in the bones, in the ligaments, or in the cartilage. The bones may be so shortened that they slip by one another in the ligaments like the ends of a flail. When the ligaments have been most affected by destruction the still healthy bones have nothing to hold them together except the soft parts. The cartilages are affected only with the other structures, and then slowly.

appearance of the joint-disease. We may in these cases suspect that the first anatomical change resulting from the central lesion was the degeneration of the joint apparatus. This produced a point of least resistance, determining the seat of the tubercular disease which first brought the patient to the surgeon. Then the other symptoms followed slowly and unrecognizably while the patient was under treatment for the joint affection.

Such a case is reported by Kummer (Deutsch. Zeitschr. f. Chir. 27, p. 7): A man of fifty died of tabes four years after the appearance of tubercular olecronitis.

The consideration of this subject leads to the following suggestions:

Central trophic disease does produce peripheral, involutionary degeneration, which determines the localization of infection in the peripheral part infected.

In all cases of joint disease, especially when painless, the possibility of a neuropathic origin should receive attention, and careful examination of the various nerve-functions should be instituted.

In all cases of multiple joint-tuberculosis, especially when symmetrical, the fear of a primary neuropathic origin renders the prognosis less favorable.

LITERATURE.

*Ziegler:* Path. Anat. Jena, 1887, p. 179.

*Gowers:* Diseases of the Nervous System. Phila., 1888, pp. 260 and 299.

*Gaskell:* The Journal of Physiology, Vol. VII, 1886.

*Balfour:* A Treatise on Comparative Embryology, 1885.

*Joseph:* Beitraege z. Lehre v. d. trophischen Nerven. Virch., Arch. CVII, p. 119.

*Rotter:* Die Arthropathien bei Tabiden. Arch. f. klin. Chir., 36, p. 1.

*Sonnenburg:* Die Arthropathia tabidorum. Do., p. 127.

*Adamkiewicz:* Ref. Virch. u. Hirsch's Jahresbericht f. 1885, Bd. I, p. 58; 1886, Bd. I., p. 60.

125 STATE STREET.

